# Enhancing youth resilience to agri-food system shocks: a pathway to climate justice

**DOI:** 10.1088/2976-601X/ae9f61

**Published:** 2026-09-24

**Authors:** Wendy Geza, Tinashe Lindel Dirwai, Nosipho Dlamini, Nafiisa Sobratee-Fajurally, Tafadzwanashe Mabhaudhi

**Affiliations:** 1 Centre for Transformative Agricultural and Food Systems, University of KwaZulu-Natal, Private Bag X01, Scottsville, 3209 Pietermaritzburg, South Africa; 2 International Water Management Institute (IWMI), 12.5 km Peg, Mt Pleasant, Harare, Zimbabwe; 3 Institute of Natural Resources, 67 St Patricks Rd, Scottsville, Pietermaritzburg 3201, South Africa; 4 Centre on Climate Change and Planetary Health, London School of Hygiene and Tropical Medicine, London, United Kingdom

**Keywords:** inclusive climate policies, equity, sustainable development, transformative adaptation, empowerment, well-being

## Abstract

Meaningful youth engagement in climate policy, resilience-building, and agrifood system transformation is vital to ensuring that young people are prioritised in national and global agendas. This review situates youth resilience within the context of climate justice, recognising that those in low- and middle-income countries (LMICs) disproportionately experience climate-related shocks due to systemic inequalities. Guided by the Preferred Reporting Items for Systematic and Meta-Analyses Protocol framework, a scoping review was undertaken to examine how youth in LMICs experience and respond to agrifood system vulnerabilities arising from climate change, economic disruptions (including COVID-19), and conflict, with a focus on education and employment outcomes. Systems thinking served as the central analytical approach to identify interdependencies, feedback loops, leverage points, and resilience pathways. The findings reveal limited research quantifying youth-specific vulnerabilities and resilience strategies within agrifood systems. While broad impacts are recognised, youth agency and adaptive capacities remain underexplored. The interactions among climate impacts, contextual vulnerability, health-productivity constraints, food insecurity and governance conditions create compounding risks that exacerbate food insecurity and social inequity among rural and urban youth. To address these gaps, the study proposes a conceptual framework that integrates climate justice principles into resilience-building strategies, promoting equitable access to resources, inclusive governance, and youth-led adaptation. By mapping interconnections across agrifood systems, climate, equity, education, and employment, the study charts resilience pathways toward socially inclusive, climate-adaptive, and economically sustainable food systems, underscoring the need for policies and research that centre youth agency and lived experiences.

## 1. Introduction

The agri-food systems literature in low- and middle-income countries (LMICs) has increasingly focused on the intersection of sustainability, resilience, and inclusive development (Alarcon *et al* 2021, Barrett *et al* 2022). Several scholars have examined the systemic pressures on these systems, ranging from climate change and resource degradation to economic shocks and geopolitical instability (Tendall *et al* 2015, Darnhofer 2021, Sperling *et al* 2022). These disruptions have prompted calls for transformative approaches that build resilience, not only at the system level but also among individuals and communities (Lloyd *et al* 2024). Within this broader discourse, youth have emerged as a critical demographic, both vulnerable to shocks and uniquely positioned to drive transformation. Studies by Townsend *et al* (2017), Phillips and Pereznieto (2019), and Sumberg *et al* (2019) highlight the potential of agri-food systems to create economic opportunities for youth. However, these opportunities are often constrained by structural barriers, limited access to resources and exposure to compounding risks (Cenacchi *et al* 2020, Geza *et al* 2021, World Food Forum 2023).

The concept of resilience has evolved across disciplines (Doherty *et al* 2019). In social-ecological systems literature, resilience is defined as the capacity to anticipate, absorb, adapt and transform in response to disruptions (Coopmans *et al* 2021). At the individual level, especially among youth, resilience can be understood as psychological, social, and economic capabilities that enable coping and recovery (Gitz and Meybeck 2012; FAO 2024). Despite increasing attention, such as the High-Level Panel of Experts report on youth engagement and employment in agriculture and food systems (Hple 2021) and the comprehensive FAO status report on youth in agrifood systems (FAO 2025)—there remains a gap in understanding how youth in LMICs specifically experience and respond to shocks within agri-food systems, and how their resilience can be strengthened through systemic interventions. Recent studies have begun to examine the impacts of climate change, economic shocks such as COVID-19, and conflict on youth outcomes, particularly in education, employment, and food security (Habtewold 2021, Ntege and Mukuna 2023, World Food Forum 2023). However, much of this work remains fragmented, with limited integration of systems thinking approaches that consider interdependencies, for example, between youth agency, resilience, and agri-food system transformation (Glover and Sumberg 2020, Dentoni *et al* 2023).

To achieve better and more positive outcomes for People and the Planet, there is a need to transform agri-food systems to make them more sustainable, resilient, equitable, and inclusive (Carter *et al* 2018, Fedele *et al* 2019, Stefanovic *et al* 2020). In doing so, investing in youth and building their resilience and agency as catalysts for agri-food systems transformation becomes crucial (Cenacchi *et al* 2020, Hple 2021, Dutta *et al* 2023).

Moreover, employment in the agrifood system plays a vital role in sustaining livelihoods in LMICs, where job creation is concentrated in sectors that require mostly low- to intermediate-skilled labour, such as agriculture, trade, and food services (ILO 2025). Youth unemployment remains significantly higher than adult rates, with regions such as North Africa, the Arab States, and Southern and Eastern Asia experiencing particularly high levels (ILO 2024, 2025). Despite a global youth unemployment rate at a 15 year low of about 13% in 2023, a large share of youth remains excluded from employment, education, or training (NEET), highlighting missed opportunities for human capital development (Ilo 2024). Thus, strengthening youth participation in agri-food systems also means building their resilience and agency, thereby enhancing their economic inclusion, well-being, and prospects. Furthermore, climate justice recognises that those least responsible for climate change—such as youth in LMICs—are often most affected by its impacts (Tafon *et al* 2025). Within agri-food systems, this injustice is compounded by inequalities that further limit youth’s access to resources, meaningful involvement in decision making and their overall adaptive capacity.

This review contributes to the literature by synthesising evidence on youth resilience in the context of agri-food system shocks, identifying coping strategies, and applying a systems-oriented framework for building youth resilience in the Global South. In addition, this review situates youth resilience within the broader context of climate justice (Newell *et al* 2021, Tafon *et al* 2025), recognising the disproportionate burden of climate-related shocks on marginalised youth in the Global South. It bridges gaps between resilience theory, youth development, and agri-food systems transformation, offering insights for policy and practice. Moreover, building youth resilience at the system and individual levels within the agri-food system translates into the realisation of several interlinked Sustainable Development Goals (SDGs) (Hple 2021, World Food Forum 2023). This review seeks to answer the following main research question: What essential resilience capabilities do young people possess to effectively handle disruptions within the agri-food system? Secondary to this, we also ask:

i. What specific strengths and abilities do young people have regarding their resilience in coping with and adapting to challenges within the agri-food system?
ii. How do young people navigate and respond to diverse shocks and disruptions within the agri-food system?
iii. What is the vulnerability and likelihood of different groups of young people experiencing these shocks?
iv. Based on current evidence, how can a systems-thinking approach be applied to identify interdependencies, feedback loops, and leverage points within agri-food systems to strengthen youth resilience in the Global South?

## 2. Methodology

### 2.1. Conceptual framing

This study builds on foundational work exploring youth participation in agri-food systems (Geza *et al* 2021, 2022, 2023) and developing pathways for youth inclusion in agri-food systems (Geza *et al* 2026). The current study advances this work by focusing on the key subject of youth resilience in agri-food systems as follows:

• The first section examines existing evidence on the types of shocks affecting youth, the factors that heighten their vulnerability or sensitivity to these shocks, and the coping strategies they employ in response to these stressors;
• The second section examines the implications of these findings for enhancing youth resilience and incorporating resilience-building approaches into the design and implementation of development initiatives that foster sustainability; and
• The final section presents a conceptual framework for enhancing resilience and mitigating youth vulnerability to shocks within the agri-food system, using a systems thinking approach across four interconnected levels.

In this study, we adopt an agri-food systems lens, which refers to the interconnected set of agricultural production systems (food and non-food) and related activities, including input provision, production, processing, distribution and associated services, along with their ecological and socio-economic interactions (Alonso-Adame *et al* 2024). Food systems, for example, intersect with the agri-food system through food production, but cover a broader range of actors and activities that extend beyond production-focused activities to include governance, diets and environmental outcomes (El Bilali *et al* 2021, Von Braun *et al* 2021). Moreover, compared to the broader food system, the agri-food systems framing highlights the links between agricultural production and food value chains, while still recognising system-wide feedbacks and dynamics (Pal and Sharma 2018).

However, agri-food systems extend beyond agriculture as a sector and beyond value chain approaches that emphasise linear flows of commodities. Value chains operate within agri-food systems and describe the sequence of activities and actors that create value through the production, transformation and delivery of products to consumers. The primary function of a value chain is to add value through physical transformation of products, process innovation, and marketing that responds to consumer demand (Pal and Sharma 2018). Agricultural value chain actors include input suppliers (e.g. seeds, fertilisers, chemicals, machinery), farm production (crops, livestock, horticulture), storage, traders and transporters, processors (e.g. food, feed, fibre), and retail and distribution (Ganeshkumar *et al* 2021).

Given the above, in the context of this review, we consider youth in agri-food systems not simply ‘young farmers’ but young people whose lives, livelihoods, and agency intersect with any part of the agri-food system (as described above). This includes young women and men who participate as workers, entrepreneurs, students, innovators, advocates, consumers, and decision-makers across the agri-food system. This framing is broader than agriculture alone, encompassing off-farm and non-farm roles linked to food value chains and food-system governance. It also recognises that youth may be engaged in farming, off-farm, or non-farm livelihoods, formally or informally, full-time or part-time, and may combine these. It is understood that youth are not a heterogeneous population; they differ based on attributes such as gender, age, educational level, skills, socio-economic context, interests and aspirations (Geza *et al* 2021, 2022). Moreover, their engagement with and aspirations within the agri-food systems vary depending on how these attributes interact. Youth participation is understood as active engagement and influence, not just presence, token representation, or informal labour (see Geza *et al* 2026).

### 2.2. Search strategy

The search strategy was based on the understanding of the terminologies and framing presented above. It, therefore, focuses on literature that uses the particular terminology. The literature review was guided by the Preferred Reporting Items for Systematic and Meta-Analyses Protocol (PRISMA-P) (Page *et al* 2021). The approach facilitates standardised, replicable evidence-based data extraction and synthesis. The search utilised keywords and associated synonyms to identify relevant literature, accounting for variations in relevant keywords across different databases (see table 1). In selecting the key search terms for the review, the aim was to focus specifically on the resilience component, using an understanding of vulnerability to better identify and contextualise the elements that contribute to resilience. This approach enabled us to more clearly distinguish resilience-related factors and emphasise their importance within the broader framework of youth risk and vulnerability in agri-food systems. Additionally, we used broad terminology for the various categories of shocks to broaden the scope of our search. For example, a search for ‘climate change’ would typically retrieve literature on weather extremes and related topics, as these are generally discussed in the context of climate variability and change.

**Table 1.** Search key terms and synonyms.

Keywords	Synonyms
Youth	Young people, adolescent*, young adults, young women, young men, young migrants, youth with disabilities
Resilience	Resilience capabilities, resilience abilities, coping skills, adaptation abilities, resilience strengths, adaptive capacity, coping strategies, vulnerability
Agri-food systems	Agriculture, food system, farming, food production, food services, non-agricultural manufacture, non-food, non-agriculture supply chains
Shocks	Disruptions, crises, challenges, vulnerabilities ▪ ‘Economic shocks’ OR ‘Economic crisis’ OR ‘food price crisis’ OR market fluctuations OR supply chain disruptions OR ‘financial crisis’ OR ‘price shock’ OR ‘financial shock’ ▪ ‘Environmental shocks’ OR ‘climate variability’ OR ‘climate change’ OR ‘pest and disease outbreaks’ OR COVID-19 ▪ Technological shocks OR digital disruptions ▪ Conflict OR civil unrest OR War OR civil unrest

Furthermore, although the review’s primary focus was on the resilience of rural youth, the literature search was open-ended to retrieve literature across all contexts (rural, peri-urban and urban) to allow for a broader comparison of socio-economic factors contributing to youth’s ability to withstand and adapt to adversities. In the context of this review, for statistical purposes, youth is defined by the United Nations as persons aged 15–24. However, it is noted that some LMICs define youth as persons between the ages of 15 and 35.

Given the breadth of research on the topic, the study applied the population, intervention, comparison, and outcome (PICOS) framework to define the search strategy. The PICOS was selected over the Sample, Phenomenon of Interest, Design, and Evaluation because of its ability to yield a high number of search hits (Methley *et al* 2014). This ensured that the study did not overlook or omit any relevant literature that could help answer the research question. The disaggregated PICO tool was used to derive search strings combined with Boolean operators (AND/OR) and singular and plural forms to form an aggregated search syntax (table 2). As no comparisons are considered, the C in PICO was used to define context, i.e. agri-food systems. The full search string and the filters applied in the databases are provided in the supplementary information (see Table SI 1).

**Table 2.** Application of the PICO tool to develop individual search strings for inputting into databases and websites.

PICO	Search strings
P	‘youth’ OR ‘young people’ OR ‘adolescents’ OR ‘young adults’ OR ‘young men’ OR ‘young women’
I	‘disruptions’ OR ‘shocks’ OR ‘challenges’ OR ‘crises’ OR ‘impacts’ OR ‘economic’ OR ‘market fluctuations’ OR ‘supply chain disruptions’ OR ‘environmental’ OR ‘climate variability’ OR ‘pest outbreaks’ OR ‘disease outbreaks’ OR ‘COVID-19’ OR ‘conflict’ OR ‘technological’ OR ‘digital disruptions’
C	‘Agri-food system’ OR ‘agriculture’ OR ‘food system’ OR ‘farming’ OR ‘food production’ agric* OR agri-food* OR agri-food* OR food* OR fish* OR aquaculture OR forest* OR livestock* OR farming OR ‘food production’ OR ‘agri?food processing’ OR ‘agri? food manufactur*’ OR ‘agri?food trad*’ OR ‘agri?food value chain’
O	‘Resilience capabilities’ OR ‘coping skills’ OR ‘adaptation abilities’ OR ‘resilience strengths’ OR ‘resources’ OR ‘assets’ OR ‘capabilities’ OR ‘skills’ OR ‘abilities’ OR ‘vulnerability’

### 2.3. Information sources

To identify relevant literature and studies, this review searched for peer-reviewed studies in the Scopus and Web of Science databases and grey literature from websites of international development organisations/institutes recognised for conducting research on agri-food systems in LMICs, namely:

• Food and Agriculture Organization of the United Nations (FAO)
• United Nations (UN)
• World Bank
• CGIAR
• Institute of Development Studies (IDS)
• International Fund for Agricultural Development (IFAD)
• CABI
• International Institute for Environment and Development (IIED)

The Web of Science and Scopus databases were selected for their ability to archive academic and scientific information. The scope of journals in these databases also covers research in science, technology, the social sciences, and sustainable development. The databases were searched from August 15–20 September 2024, and an updated literature search was conducted in November 2025. Once the search was completed, a literature database was shared with a panel of experts from the FAO Agri-food Economics and Policy Division (ESA) to identify key documents that the search strategy might have missed. The panel identified one additional document, which was added to the review, and no further documents that met the inclusion and exclusion criteria were retrieved in the updated search.

### 2.4. Eligibility criteria and critical appraisal

An eligibility criterion was used to assess the suitability and relevance of identified studies for inclusion in the review. Although the review has a global focus, it emphasises youth issues in LMICs and youth in rural economies. It searched for studies conducted since 2015, after the adoption of the SDGs and the Paris Agreement on climate change. A two-step literature screening criterion was adopted. The first step involved screening the literature against the inclusion and exclusion criteria (see table 3). Studies that did not meet this criterion were excluded from the review. In the second step, the quality and strength of evidence were assessed for studies considered for review by two reviewers using the JBI critical appraisal checklist for qualitative research (Lockwood *et al* 2015). The critical appraisal aimed to evaluate the methodological quality and potential bias of the included studies. Rather than excluding studies based on quality, we used the appraisal to inform the rigour and robustness of the synthesis by weighting them. Findings from higher-quality studies were prioritised during theme development, whereas themes supported primarily by lower-quality studies were treated cautiously.

**Table 3.** The inclusion and exclusion criteria for screening literature.

Criteria	Inclusion	Exclusion
Language	English	Any other language other than English
Date	2015—present	Research conducted before 2015
Location	Low- and middle-income countries	Research conducted in high-income countries
Subject	Youth and agri-food Systems	Research conducted on other societal groups in or outside the agri-food systems
Type of article	Peer reviews research, opinion papers, technical reports, working papers and book chapters	Reviews that only summarise the existing literature, thesis and dissertations

### 2.5. Data extraction and synthesis methods

Data were sought on categories of shocks that impact young individuals within the agri-food system, including the diverse tangible and intangible resources available to navigate and respond to these challenges. Specifically, the review classified outcomes and data items on:

a) Various shocks impact young individuals within the agri-food system.
b) The diverse tangible and intangible resources at their disposal to navigate and respond to these challenges (coping strategies)
c) The vulnerability of youth and their likelihood of experiencing shocks.

The data were analysed using mixed-methods, including bibliometric analysis (RStudio) and content analysis (NVivo). Bibliometric analysis was crucial for mapping the evolution of research on youth and resilience, both spatially and temporally, as well as for conducting network and thematic analyses and identifying trends in the discourse (Lim *et al* 2024). The content-based analysis facilitated insights into the youth and resilience discourse, as well as the mapping of evidence and identification of gaps in the literature. The R-studio script facilitated the (1) tokenisation of the text data, (2) defining keywords for the defined shock absorbance themes, and (3) categorising thematic words and counting occurrences of various types of shocks. NVivo was used for content analysis to identify patterns and themes related to youth vulnerability and the likelihood of experiencing these shocks. Additionally, it provided context on the occurrence and resilience of youth to various types of shocks.

### 2.6. Systems thinking approach and the use of causal loop diagramming (CLD)

A systems-thinking approach was used to conceptualise an actionable pathway for strengthening youth resilience within the agri-food systems of the Global South. This approach facilitates an analysis of how individual elements interact to perform specific functions by revealing patterns of interaction and behaviour that may contribute to recurring challenges (Haines 2000, Arnold and Wade 2015, Burton 2021). Systems thinking enables a comprehensive understanding of complex problems and of how particular actions affect various components, both independently and collectively (Meadows 2008). Tools such as systems maps, particularly CLDs, can effectively illustrate the interconnectedness of system elements, identify feedback loops, explain system behaviour over time, and highlight leverage points that may lead to transformative change (Turner *et al* 2016).

Drawing on content and bibliometric analyses, we identified key themes and coping strategies employed by youth to manage shocks and vulnerabilities across individual, community and organisational levels. These themes were subsequently analysed and synthesised to generate insights that informed the development of a conceptual resilience framework. The framework highlights the principal resilience strategies used by youth to cope with diverse shocks and disruptions across multiple levels of influence. In addition, an agri-food system perspective was incorporated as an overarching layer to strengthen youth capacities for managing shocks and vulnerabilities. Framed within a climate justice perspective, the framework identifies strategic entry points from literature for enhancing resilience through targeted investments and interventions across all levels (e.g. climate education, skills development, and income diversification at the individual level). By strengthening youth’s capacities to anticipate, absorb, adapt to, and transform in response to evolving risks, the framework outlines a pathway for reducing vulnerability and promoting long-term resilience.

Building on these thematic findings, we developed an evidence-informed casual influence map to synthesise the directional and reciprocal relationships identified across the reviewed literature. The map highlights three interconnected pathways: (a) climate impacts, contextual vulnerability, resource access and food insecurity; (b) physiological heat stress, mental health burdens and food-system productivity; and (c) governance conditions, conflict, systemic COVID-19 impacts and food insecurity. Building from the casual influence map, a CLD was developed to highlight the intricacies and influence of additional factors such as gender, locality, educational level and disability that further heighten youth vulnerability. These additional factors emanate from the thematic analysis. Together, these maps illustrate the complex interactions through which shocks and vulnerabilities influence youth resilience within agri-food systems. To further explore these dynamics, a second CLD was developed to hypothesise feedback relationships amongst the interventions and capacities in the proposed resilience framework and to unpack interconnections amongst identified issues, highlighting additional insights into the outcomes of coping strategies. The causal maps were created using the Stella software.

## 3. Results

### 3.1. Literature search

**Figure 1.**
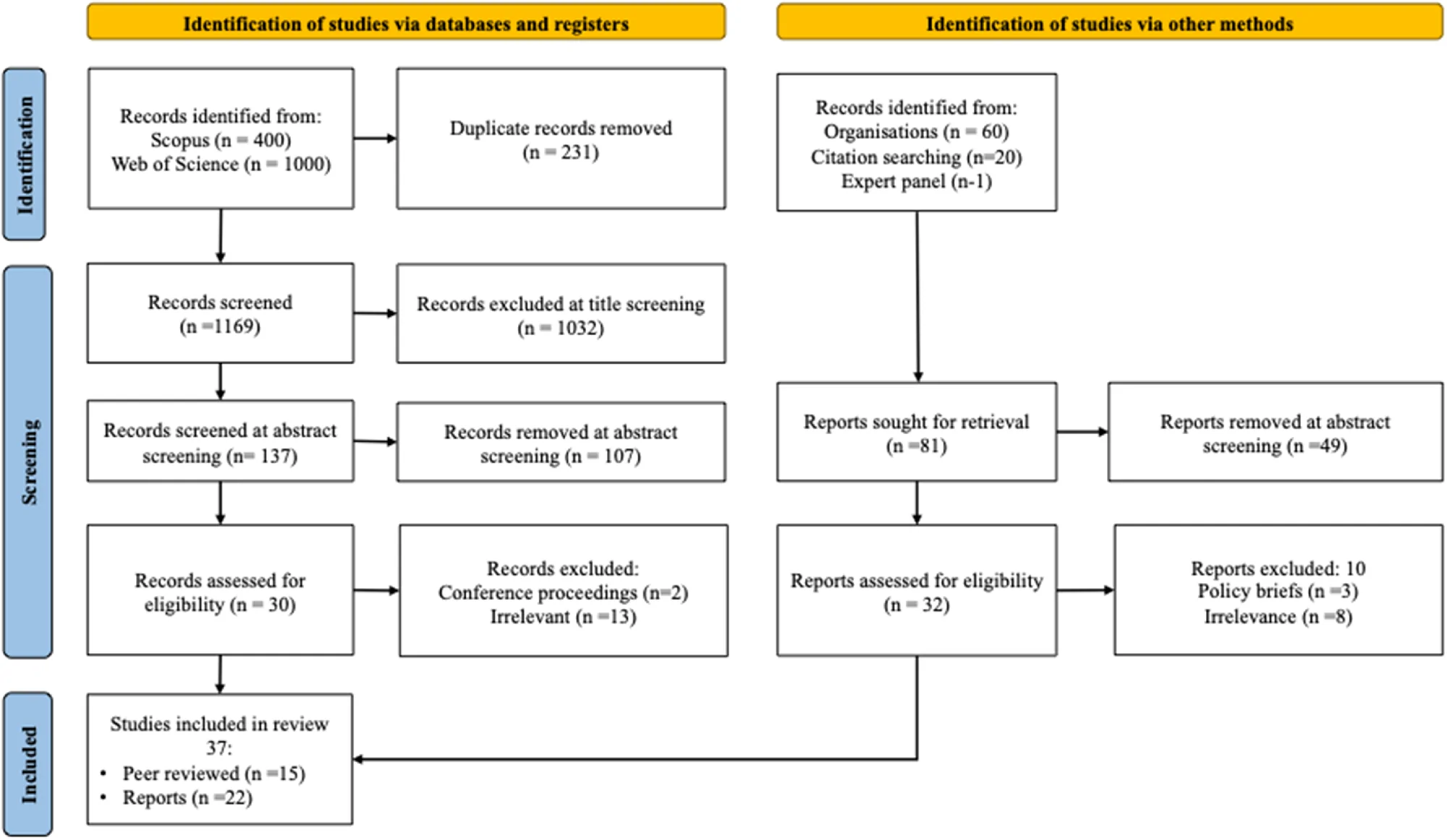
PRISMA flow diagram for the review outlining the searches of databases, registers and other sources (Page *et al* 2021).

The initial search generated a total of 1481 documents, which included peer-reviewed research (1400), grey literature (80), and one report identified by the panel of experts (see figure 1: PRISMA flow diagram). As a result, 231 duplicates were removed, and the abstracts of the remaining articles were screened against the inclusion and exclusion criteria. A two-step literature screening criterion was adopted. Firstly, at the full-text screening stage, 25 articles were removed because they did not meet the inclusion and exclusion criteria. Additional articles were excluded because they were irrelevant to the study’s objectives or lacked sufficient data. Examples of articles that were excluded are literature reviews (Ncube *et al* 2024), policy reviews and policy briefs (Chamberlin and Sumberg 2021, Sumberg 2021, Thorsen and Koffi 2021, Huambachano *et al* 2022), book chapter (Mnimbo *et al* 2019) and reports that gave a general picture of the situation of rural youth in developing countries, not in the context of youth resilience in agri-food systems (Guiskin *et al* 2019, Phillips and Perzenieto 2019, Stecklov and Menashe-Oren 2019). Other articles were not relevant to the study objectives and assessed factors such as youth perceptions of agriculture (Baloyi *et al* 2023), implications of urban growth on youth migration decisions (Amare *et al* 2021) and others evaluated the impacts of youth agricultural programs and initiatives (Dickson-Hoyle *et al* 2018, Phiboon *et al* 2019, Ogunmodede *et al* 2020), see supplementary information *Tables Si 2*. Following the initial screening process, 37 articles were selected for review. In the second step, the quality and strength of evidence were assessed for studies considered for review by two reviewers using the JBI critical appraisal checklist for qualitative research (Lockwood *et al* 2015). The studies included in this review were of good quality, and no significant issues were noted in data collection, analysis or bias in how the results of the included studies were presented.

### 3.2. Literature characteristics and evidence mapping

Although the articles considered for inclusion in the review met the criteria, the appraisal indicated that many did not directly address the study’s research questions. Most mentioned keywords and terms related to the search keywords, but did not directly address the research questions. A full list of studies excluded at full-text screening, along with the reasons for exclusion, is presented in the supplementary information (Table SI 2). The evidence mapping was based on the 37 articles included in the review. However, of the 37 articles, only four reported evidence of youth resilience to shocks and vulnerabilities in the agri-food system, as well as their coping strategies (Baliki *et al* 2019, FAO 2024, Nigussie *et al* 2024, Shams *et al* 2024). An additional five articles vaguely reported disruptions affecting youth in the agri-food system, such as drought, climate distress and mental health, communication disruptions and suspension of a mentorship program during COVID-19 and abuse amongst smallholder youth farmers (Baliki *et al* 2019, Kabbani 2019, Prencipe *et al* 2023, Kaki *et al* 2024, Kitole *et al* 2024). Moreover, this study focused on specific shocks, including climate change, economic shocks (such as COVID-19), and conflict. In many of the included studies, these terms highlighted the broader global challenges affecting the agri-food system, particularly in the introduction and literature review sections.

For papers published after 2020, the effects of COVID-19 were also broadly discussed in the introduction, literature review, and discussion sections, focusing on the pandemic’s general impact on the agricultural industry and working hours, e.g. Unnikrishnan *et al* (2022). Climate change and resilience are cross-cutting themes in the agri-food system. Keywords such as resilience (including descriptions and associated terms, e.g. resilience capabilities, coping strategies, adaptation) and shocks were mentioned as ‘buzz words’ linked to broader climate change adaptation and mitigation strategies in agri-food systems. No actual data were collected to explicitly operationalise resilience capacities (i.e. anticipate, absorb, adapt) in the context of youth, or to quantify youth impacts or responses. However, it was broadly acknowledged that young people in the agri-food system would be affected by climate change and strengthening their resilience is imperative, as noted by Townsend *et al* (2017) and Faith *et al* (2020). There is limited evidence to quantify and attribute the impacts of various shocks and vulnerabilities in the agri-food system on youth, the extent of those impacts, and how youth anticipate, absorb, cope with, and/or adapt to them. Based on the characteristics of 37 studies included in the review, the current body of literature lacks evidence on youth resilience to shocks and vulnerabilities in the agri-food system. This could be a direct outcome of the positioning of current strategies and initiatives. They are primarily aimed at increasing youth engagement and participation in agri-food systems, suggesting that youth are currently underrepresented in these systems. Hence, the primary research focus is not yet on their resilience. The primary areas of interest in the literature are the following, which were not directly linked to the study’s research objectives.

a) Identification and quantification of barriers related to youth participation in agriculture from a perspective of assets, skills and lack of structural transformation (Trevor and Kwenye 2018, Loga *et al* 2022, Khamis *et al* 2023, Kaki *et al* 2024).
b) Description of youth typologies, the landscape of opportunities existing in those settings, the influence of various factors (e.g. youth asset and capability profile, socio-cultural context and the economy) and the political landscape on employment opportunities (Arslan *et al* 2019, Stecklov and Menashe-Oren 2019, Sumberg *et al* 2019, Trivelli and Morel 2021).
c) Entrepreneurship and evaluation of policy programmes targeted at encouraging participation and capacity building for youth in the AFS, comparing participants and non-participants of these programs (Bello *et al* 2021, Unnikrishnan *et al* 2022, Poungchompu and Phuttachat 2023).

Supporting youth and building their adaptive capacity, particularly those in LMICs, will remain a formidable task without understanding the types of risks they face and the levels of exposure and vulnerability across economic, social, geographic, and environmental factors. Thus, there is a need to conduct proper risk and vulnerability assessments focused on youth and agri-food systems across their various contexts, as exposure and vulnerability can vary by context. Moreover, it is critical in unlocking and delivering positive agri-food system outcomes for youth in LMICs and attaining SDG 1 (end poverty), 2 (zero hunger), 3 (good health and well-being), 5 (gender equality), 8 (decent work and economic growth), 10 (reduced inequalities), and 13 (climate change action).

Much of the research has focused on understanding youth participation in agri-food systems, examining perceptions, barriers, and opportunities from the perspectives of assets, skills, socio-economic factors, and the landscape of opportunities for youth in various typologies. Although this is important, the reality is that the current generation will experience increasing impacts of climate change, recurring shocks, and risks that will amplify existing vulnerabilities. Therefore, building a robust evidence base and understanding youth’s exposure, vulnerability and risks to such shocks, particularly climate change exposure, is crucial to building a future resilient agri-food system (Faith *et al* 2020).

### 3.3. Bibliometric analysis: evolutionary/thematic mapping

**Figure 2.**
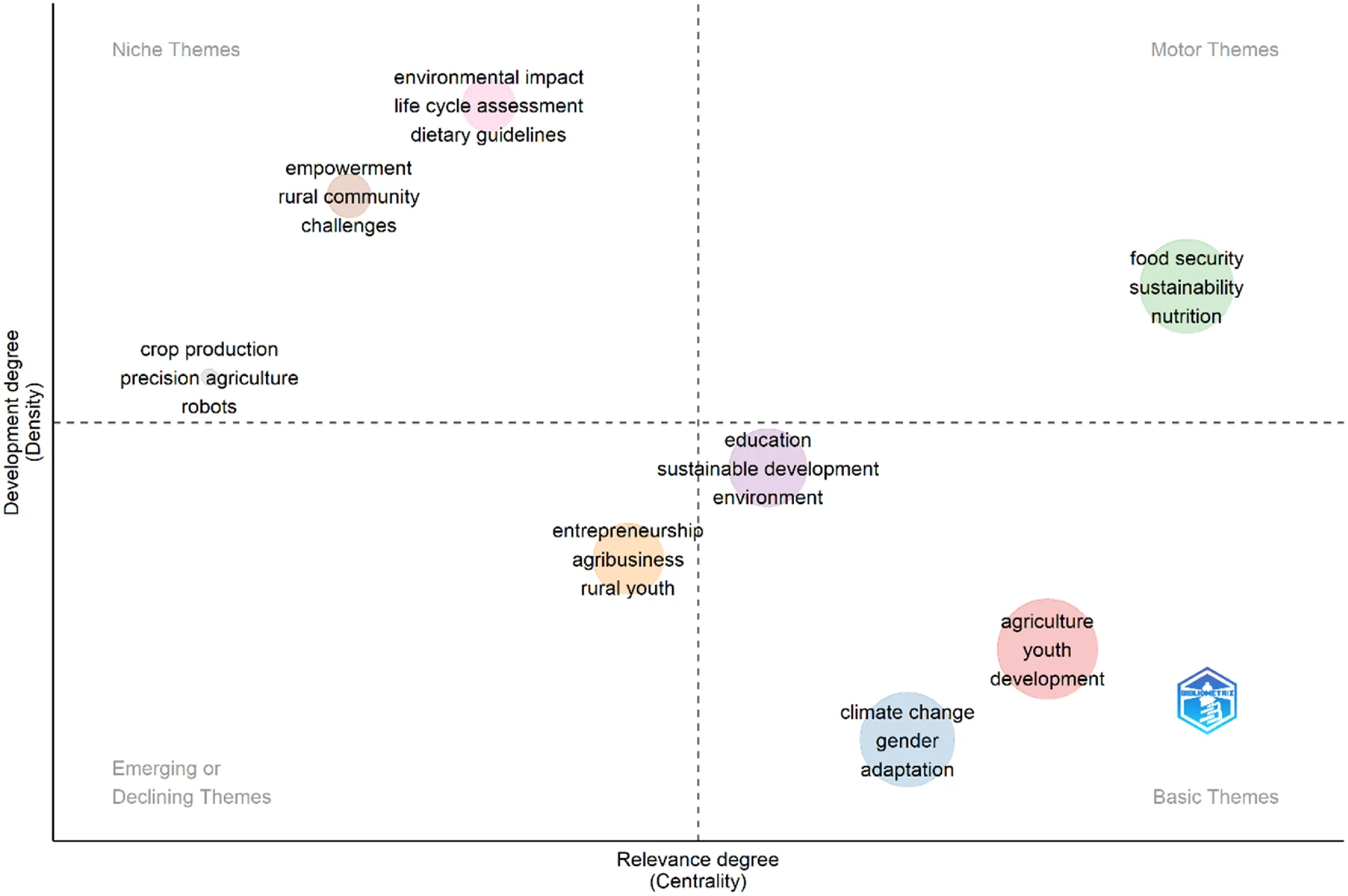
Thematic map showing the concentration of research clusters in four quadrants. The upper-left quadrant (niche themes) contains specialised but less influential themes. In contrast, the upper-right quadrant (motor themes) includes well-developed, highly relevant topics such as food security and sustainability. The lower-left quadrant (emerging or declining themes) highlights newer or less-developed areas, such as agribusiness, entrepreneurship, and rural youth. The lower-right quadrant (basic themes) represents broad, foundational issues—climate change, gender, youth, and development—that are central yet still evolving. The map reveals a limited focus on youth resilience, indicating an opportunity to expand and connect emerging topics with broader sustainability and development goals.

The thematic map (see figure 2) highlights a notable gap in the literature regarding youth resilience to shocks and vulnerabilities within the agri-food system. The dominant focus of existing research, located in the basic themes quadrant, centres on cross-cutting issues such as climate change, adaptation, gender, education, youth development, and sustainable development. These themes represent foundational and interdisciplinary pillars in agri-food systems research and are commonly framed in relation to the SDGs. However, while these themes are central to the discourse, their low density on the map suggests that they remain conceptually underdeveloped or fragmented, particularly in their engagement with young people’s lived experiences and adaptive capacities.

Importantly, themes such as rural youth, agribusiness, and entrepreneurship appear in the emerging or declining quadrant. This positioning suggests that these topics represent areas of inquiry that are growing or in transition, gaining traction but still lacking a strong, cohesive knowledge base. According to Donthu *et al* (2021) and Lim *et al* (2024), such themes are often poised to evolve into core research areas, provided they are further developed and integrated into broader discourses. At present, however, the primary research focus appears to be on increasing youth participation in agribusiness and entrepreneurship or on unpacking the structural challenges facing rural youth rather than explicitly examining the resilience capabilities of youth in response to system-level shocks. If these emerging themes are not further theorised and empirically supported, particularly through the lens of resilience, they risk losing relevance and fading from scholarly focus. In contrast, the motor themes of food security, sustainability, and nutrition represent well-established and highly developed research areas. Integrating youth resilience into these dominant themes can enhance the understanding of youth food and nutrition security under climate stress and increase the relevance of youth-focused policy initiatives.

**Figure 3.**
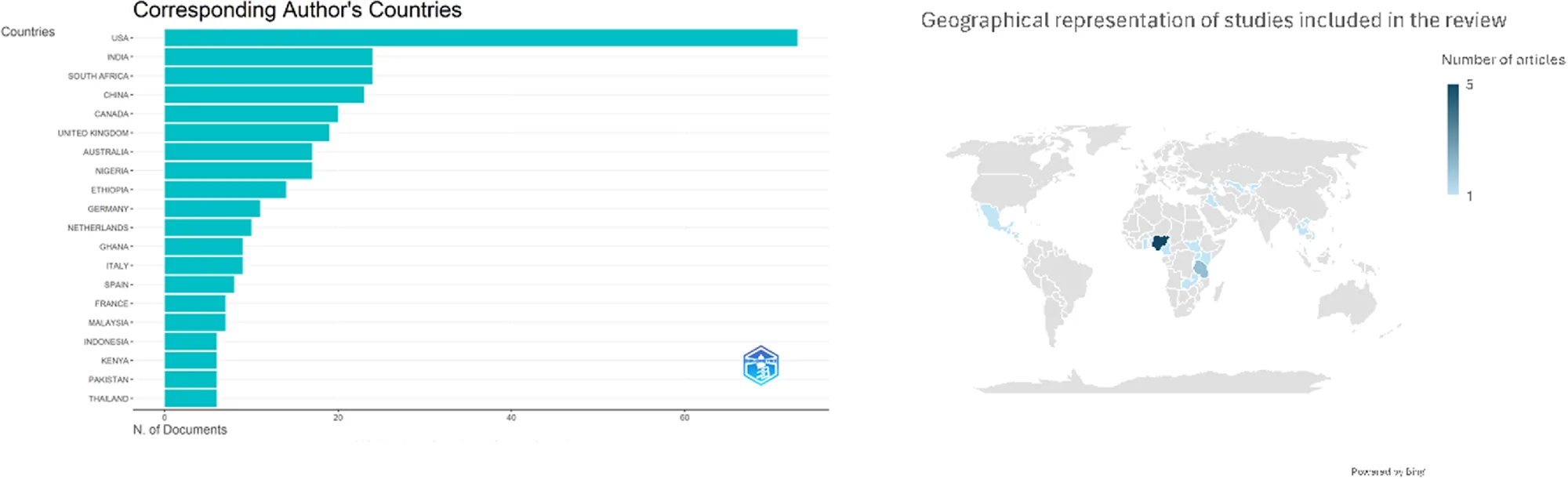
An overview of corresponding authors and their country of residence in the studies included (left) and the geographical representation of countries where data were collected (right).

**Figure 4.**
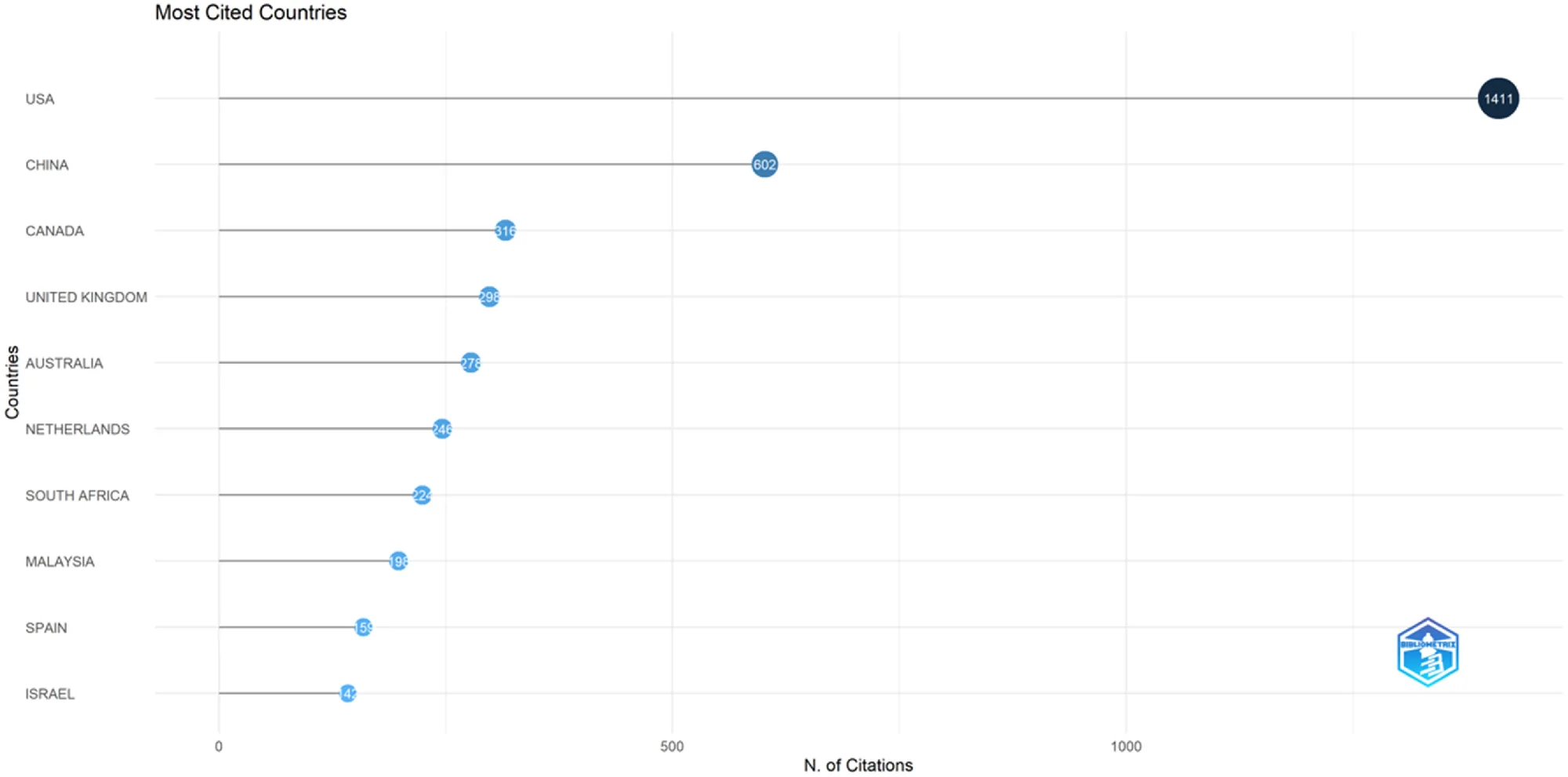
Global trends of the most cited countries in publications on youth resilience and shocks, and the subsequent evidence of their effects and coping strategies.

A total of 18 studies included in the review were conducted in a single country, and the majority were conducted in Nigeria (5) (Adelakun *et al* 2019, Bello *et al* 2021, 2022, Nigussie *et al* 2024) (figure 3). The remaining studies were conducted at a regional level, for example, in sub-Saharan Africa (Yeboah *et al* 2019), Asia and the Pacific region (Briones 2019) and others included various regions from the Global South (Arslan *et al* 2019, Dolislager *et al* 2019, Stecklov and Menashe-Oren 2019, FAO 2024). However, there is a disparity between the regions where the corresponding authors are from and where the research is conducted (see figure 3). A significant portion of the corresponding authors comes from the Global North. Moreover, examining the geographical location of the most cited documents in the studies included in the review (see figure 4), one can observe that the majority come from developed nations. This indicates a dominance of evidence from a Global North perspective, where research is being conceptualised, and further questions the relevance of these perspectives for solving local problems in the Global South, particularly for building resilience against shocks and vulnerabilities in the agri-food system. This raises concerns about the extent to which local knowledge, perspectives, and the lived experiences of youth in agri-food systems are contextualised and documented.

### 3.4. Evidence of shocks, factors increasing vulnerability and coping mechanisms

**Figure 5.**
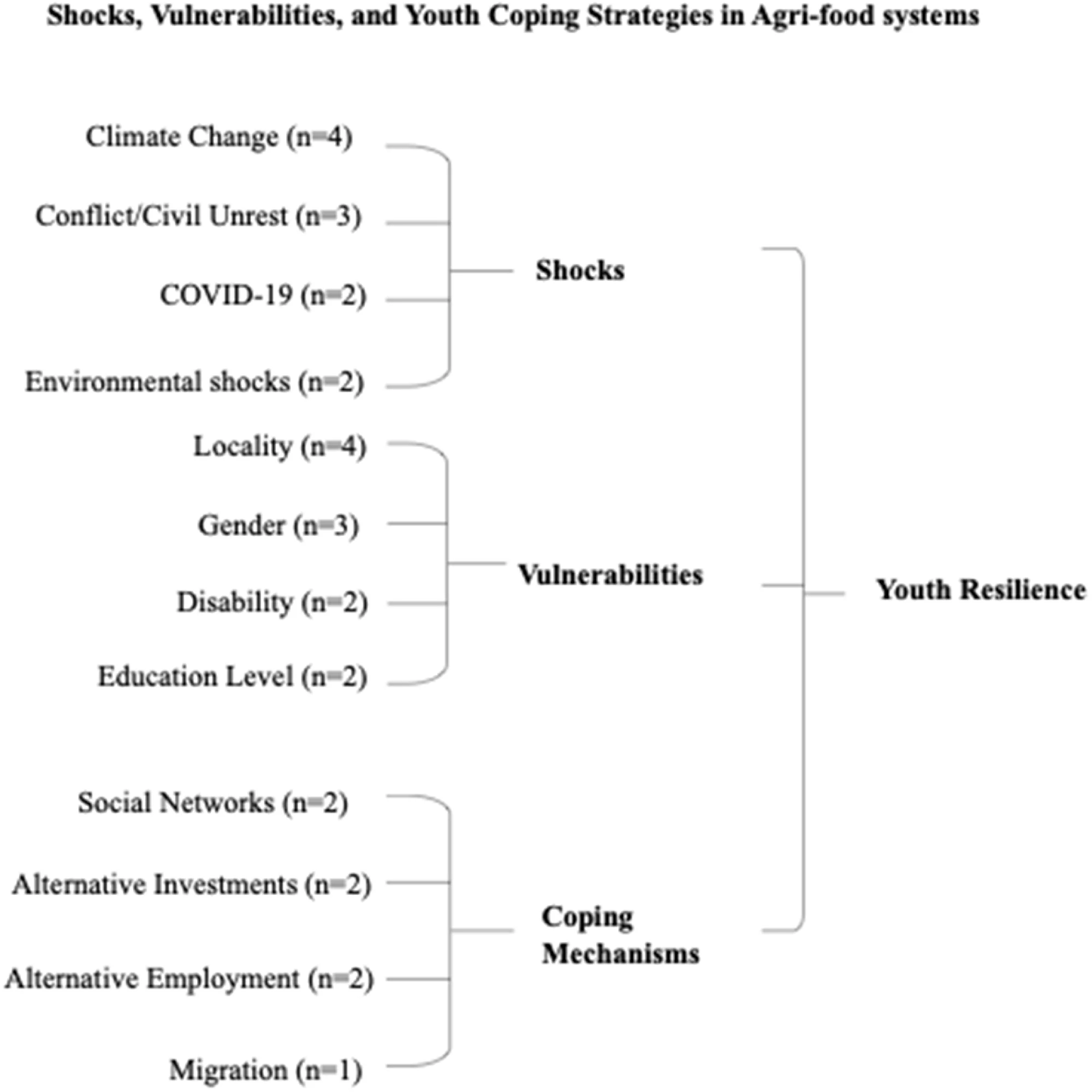
An overview of the types of shocks, factors increasing vulnerability and coping mechanisms reported by articles included in the review. This evidence map was based on the 37 articles included in the review; however, not all 37 articles reported on resilience aspects. The numbers indicate how many articles reported on each aspect of resilience. These findings underscore the climate injustice faced by youth in LMICs, where intersecting vulnerabilities—such as locality, gender and disability—amplify the impacts of climate-related shocks.

This section outlines the reported evidence on shocks affecting youth (3.4.1), factors that increase their vulnerability (3.4.2), and the diverse tangible and intangible resources at their disposal to navigate and respond to these challenges (coping mechanisms) (3.4.3), based on articles included in the review (refer to figure 5).

#### 3.4.1. Various shocks impacting youth within the agri-food system

**Figure 6.**
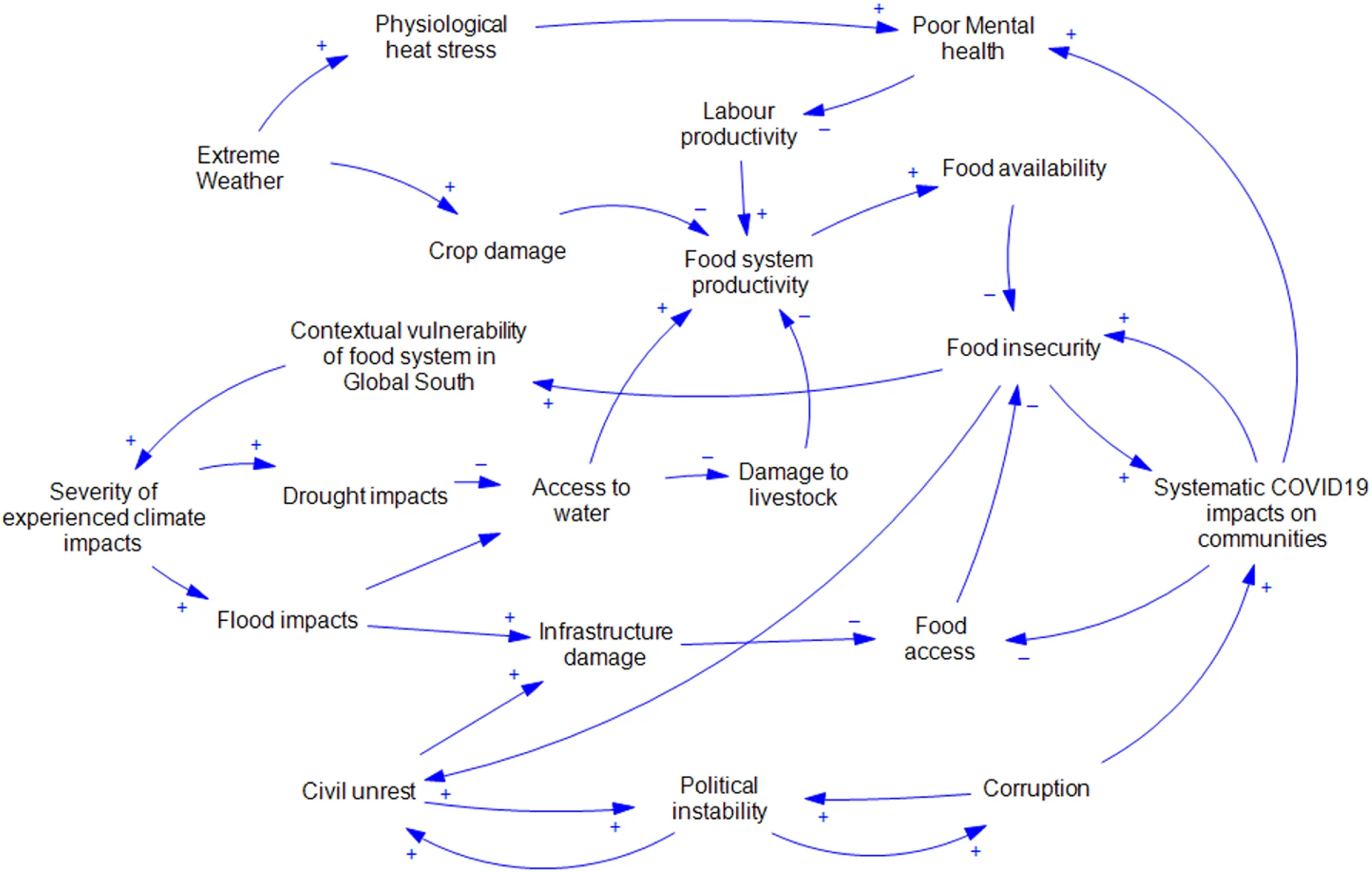
Evidence-informed causal influence map showing directional and reciprocal relationships among climate impacts, contextual food-system vulnerability, resource access, health-productivity constraints, food-system productivity, food insecurity and governance conditions in the Global South. A positive sign (+) indicates that the affected variable is expected to change in the same direction as the influencing variable, whereas a negative sign (−) indicates a change in the opposite direction, all else being equal. The map highlights three interacting pathway domains: climate impacts, resource access, and food insecurity; physiological heat stress, mental health burden, and productivity; and governance conditions, conflict, and food insecurity. The map synthesises relationships identified across the included studies and the authors’ systems interpretation. It is qualitative and should not be interpreted as a parameterised or empirically validated dynamic model.

The impacts of climate change, as shown in figure 6, combine gradual changes over time with sudden, disruptive events, and youth in agri-food systems are affected by both. The studies included in the review primarily reported on sudden, extreme weather events, such as droughts and floods (*n* = 3). One study examined the mental health implications for youth working in extreme temperatures (Prencipe *et al* 2023), by researching climate-sensitive risk factors related to livelihood activities (farming, caring for livestock, collecting water, working in extreme temperatures or near or on a body of water) and living conditions (food and water insecurity, exposures to floods or droughts and crop or livestock disease). However, no coping mechanisms were mentioned, and no data were collected to quantify the effect of climate change on youth agricultural activities (Kabbani 2019, Prencipe *et al* 2023, FAO 2024, Shams *et al* 2024). One study highlighted that environmental and climate change-related shocks are exacerbated for youth in areas with challenges related to poor infrastructure and limited access to water resources (or inadequate irrigation). Moreover, additional issues related to governance, corruption and mismanagement aggravate food insecurity (Shams *et al* 2024).

Three studies focused on the impact of armed conflict and civil unrest on youth, emphasising the disruptions it causes by destroying and damaging infrastructure, as well as its implications for food production and distribution. These studies were conducted in conflict hotspots in South Sudan, Iraq and other countries listed in the Armed Conflict Location Event Dataset (Baliki *et al* 2019, Mabiso and Benfica 2019, Shams *et al* 2024). The impact of COVID-19 was mentioned in two studies, where it disrupted communication and meetings in a mentorship program for youth in agriculture (Kaki *et al* 2024) and examined the pandemic’s impact on increasing food insecurity and depression among young people (Prencipe *et al* 2023).

The reported environmental shocks (*n* = 2) relate to climate variability and the disruptions it causes to agricultural activities, including crop damage, livestock losses, and the depletion of fish stocks, which affect food production (Macneil *et al* 2017). Moreover, these environmental challenges have also been reported to decrease food availability and accessibility and increase reliance on food imports. A systems thinking perspective reveals that climate change, its effects on mental health and productivity, and governance instability in conflict-affected areas interact to create compounding risks for youth in agri-food systems (see figure 6). These interconnected shocks exacerbate food insecurity and youth vulnerability. For example, extreme weather events increase physiological heat stress, which leads to poor mental health and reduced labour productivity. While poor governance and civil unrest undermine infrastructure and food production.

#### 3.4.2. The vulnerability of youth and their likelihood of experiencing shocks

**Figure 7.**
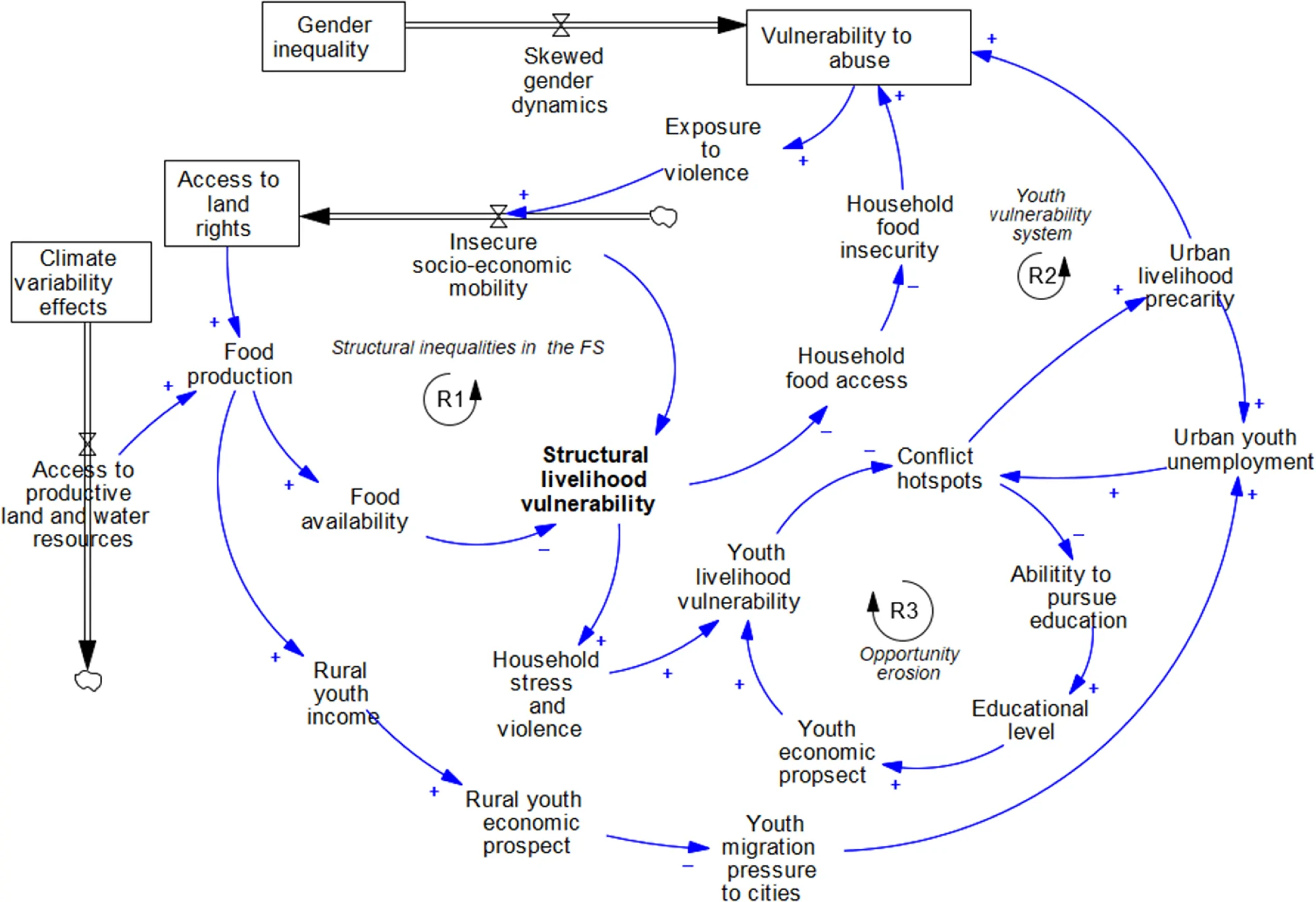
Cross-scale interactions show how gender inequality and skewed gender dynamics feed into vulnerability to abuse, which worsens food insecurity and socio-economic mobility constraints. Meanwhile, climate variability disrupts access to productive land and water resources, reducing food production and intensifying both structural and youth-specific vulnerabilities. Together, these dynamics reveal how climate stressors, gendered risks, and systemic inequalities converge to produce entrenched food insecurity across rural and urban youth populations. R1: (Structural inequalities in the FS): structural livelihood vulnerability → household stress and violence → youth livelihood vulnerability → conflict hotspot → urban livelihood precarity → vulnerability to abuse → exposure to violence → insecure socio-economic mobility → structural livelihood vulnerability. R2: (Youth vulnerability system): vulnerability to abuse → exposure to violence → insecure socio-economic mobility → access to land → food production → rural youth income → rural youth economic prospect → youth migration pressure to cities → urban youth unemployment → urban livelihood precarity → vulnerability to abuse. R3: (Opportunity erosion): Youth livelihood vulnerability → conflict hotspots → ability to pursue education → educational level → youth economic prospect → youth livelihood vulnerability.

Additional key factors significantly heighten youth vulnerability to shocks in agri-food systems, highlighted in figure 7, include gender, locality, education level, and disability. From a systems-thinking perspective, these cross-scale interactions compound and reinforce vulnerabilities, ultimately deepening food insecurity and constraining socio-economic mobility. Socio-cultural norms that limit women’s rights to access and use land for production make them more vulnerable during crises (Nigussie *et al* 2024). Female-headed households experience significantly greater income losses during extreme weather events than their male-headed counterparts (FAO 2024). In areas affected by conflict, females have increased exposure to sexual abuse and violence (Baliki *et al* 2019). Youth in rural areas are disproportionately affected by shocks and vulnerabilities such as conflict, extreme weather and violence in the AFS in comparison to their urban counterparts. Moreover, the severity of the impact is increased in cases where youth have no formal or low educational attainment, as this limits their socio-economic prospects (Baliki *et al* 2019, Mabiso and Benfica 2019, FAO 2024, Kitole *et al* 2024, Nigussie *et al* 2024).

Only two studies (FAO 2024, Kitole *et al* 2024) mentioned individuals with disabilities. Kitole *et al* (2024) reported on the vulnerability of youth in agriculture with disabilities such as blindness, hearing, physical disabilities and conditions like albinism. These youth were more vulnerable to abuse and violence, had fewer opportunities and had lower economic outcomes. In comparison, the FAO’s (2024) Unjust Climate Report highlighted the lack of available data for vulnerable groups such as Indigenous communities or individuals with disabilities. This highlights the importance of studying group factors that increase youth vulnerability to experiencing shocks in the AFS, particularly gender, disability, and locality. Youth with disabilities, special needs and youth from indigenous communities face additional challenges that are often overlooked and not accounted for, consequently limiting the presence of targeted investments in AFS.

Additionally, youth residing in volatile conflict hotspots and areas with active military operations operate under distinct economic and socio-cultural conditions that increase their vulnerability to shocks and disruptions. Their coping mechanisms and adaptation strategies necessitate responses that address a range of needs, including food access and availability during conflict, and rebuilding and strengthening their agribusiness operations post-conflict. Youth are a heterogeneous group in society, and their engagement and vulnerability in the agri-food system are diverse (Declich *et al* 2022, Madende *et al* 2023). The heterogeneity in how shocks are experienced, as well as exposure and vulnerability level. This calls for evidence to be disaggregated at local scales to better reflect the local experiences and perspectives of youth in the Global South.

#### 3.4.3. Tangible and intangible resources at their disposal to navigate and respond to these challenges (coping mechanisms)

The main strategies reported that youth use to cope with shocks and vulnerabilities in the agri-food system include reliance on social networks, selective investments, seeking alternative employment, migration and change in mode of communication (online) during the pandemic (refer to figure 5). During times of uncertainty, youth rely on social networks for financial support through loans or gifts, as well as assistance from farmer associations and government-funded projects, to mitigate risks (Nigussie *et al* 2024). Youths in conflict-affected areas cope by reducing meal frequency, diversifying their diets by incorporating wild foods, and relying on humanitarian aid and external support. Common challenges, such as food insecurity, are addressed at a community level by sharing tools and resources (e.g. seeds) (Shams *et al* 2024).

To manage the risks posed by financial constraints, youth from resource-poor households selectively invest in livestock or engage in off-farm activities (processing, distribution, and marketing). Off-farm activities are less volatile, require less capital, and yield faster financial returns than on-farm activities (Nigussie *et al* 2024). Youth-headed households intensify their agricultural activities and acquire livestock to secure food and income in a crisis. In rural areas, livestock typically serves multiple functions, including providing food and income and acting as a store of value, thereby enabling people to better cope with future stressors (FAO 2024). In addition, youth also sustain themselves by seeking alternative employment, such as casual jobs, small-scale trading, off-farm economic opportunities, and other forms of ‘hustling’ during a crisis (Shams *et al* 2024). In conflict-affected areas, young people often migrate to urban areas to pursue economic opportunities. Families also prioritise food over other paid services, such as children’s education (Baliki *et al* 2019). This emphasises the importance of off-farm activities in LMICs as a strategy to minimise the impacts of market and climate-related shocks on youth livelihoods, reduce their vulnerability and build their resilience. Moreover, under a changing climate, strategies for reducing the vulnerability of youth and increasing their resilience to shocks and risks need to be holistic and integrate (i) social protection programs to provide support and aid, (ii) training and skills development for youth to adopt diversified income-generating activities and climate-smart agricultural technologies, and (iii) leverage on existing social networks and cooperatives to share knowledge, pool resources, and collective bargaining.

## 4. Discussion

This study reveals a significant gap in the documented evidence on how shocks and vulnerabilities in agri-food systems affect youth—particularly regarding their exposure, responses, and resilience strategies. Current initiatives tend to focus on increasing youth participation in agri-food systems, often overlooking the fact that many youth are already engaged yet face substantial risks. Youth are affected by both gradual (e.g. climate change) and sudden (e.g. extreme weather) shocks, with impacts compounded by intersecting vulnerabilities such as socio-economic status, gender, disability, and political context, leading to disruptions in livelihoods and food security. Common coping strategies among youth include leveraging social networks, diversifying income sources, migrating, and making selective investments.

Our study echoes observations made by scholars such as Béné and Devereux (2023) and De Pinto *et al* (2023), who critique the widespread use of the term‘resilience’ in development discourse, often without substantive meaning or measurable indicators. As highlighted by De Pinto *et al* (2023), resilience has become a ‘buzzword’ especially in the first decade of the twenty-first century, frequently appearing in academic literature, political discussions, and project proposals, yet rarely accompanied by clear metrics or integration into monitoring and evaluation frameworks. This underscores the need for greater rigour, scrutiny, and intentionality to ensure resilience is meaningfully embedded in the design and implementation of development initiatives that promote sustainability.

Over the past decade, various scholars (Swarts and Aliber 2013, Bezu and Holden 2014, Manalo Iv *et al* 2019, Chellattan Veettil *et al* 2021, Alrawashdeh *et al* 2023, Ninson and Brobbey 2023) have examined the factors influencing youth participation in agri-entrepreneurship in LMICs. Youth often enter the sector informally, facing limited job security, inadequate institutional support, and a host of challenges that heighten their vulnerability to the shocks and disruptions inherent in food systems. Beyond informal engagement, youth face additional layers of marginalisation—including economic exclusion, gender-based discrimination and challenges, and, in some cases, disability-related harassment and abuse. These vulnerabilities are shaped by a complex interplay of micro-level factors across physical, social, environmental, economic, and institutional domains, all of which influence youth participation, well-being, and empowerment in agri-food systems (Geza *et al* 2021, 2022). Addressing these challenges requires more than facilitating youth entry into agriculture—it demands sustained support for their survival, success, influence, and long-term sustainability. This means investing in their resilience and agency to enhance educational outcomes (both formal and informal), employment opportunities, and overall well-being. Moreover, a climate justice lens further compels us to move beyond technical resilience solutions and address structural drivers of youth vulnerability. Such investments must be equitably distributed across these critical areas to ensure lasting impact. Multi-stakeholder partnerships—spanning households, communities, local and regional actors, and national and international institutions play vital roles in supporting youth resilience. These efforts must be coordinated to be effective.

Strategies must go beyond mere participation to reduce vulnerability and enhance resilience (FAO 2025). A comprehensive systems thinking approach is needed, one that centres youth empowerment, resilience-building, and agency. This calls for a multi-level strategy that addresses both short-term sensitivities (the ability to anticipate and absorb shocks) and long-term adaptive capacities (the ability to adapt and transform). Resilience-building efforts should be implemented across individual, community, and organisational levels to ensure sustained improvements in youth resilience over time.

## 5. A conceptual framework for building resilience and reducing youth vulnerability to shocks in the agri-food system

**Figure 8.**
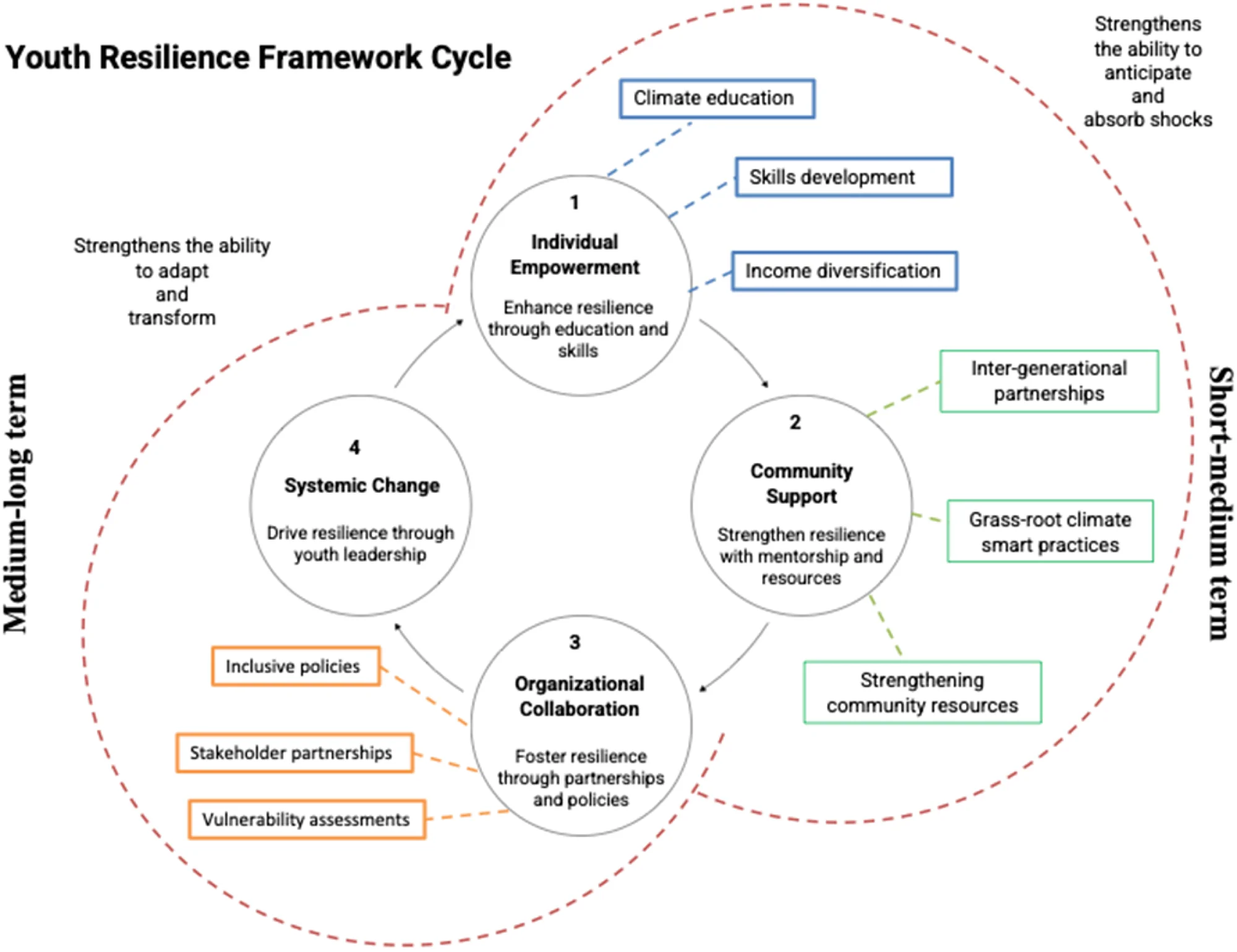
This evidence-informed conceptual framework is grounded in insights generated through thematic content analysis. It synthesises the key findings on youth resilience strategies across multiple levels (individual, community, organisational, and agri-food system). The framework not only captures the mechanisms by which youth currently navigate and respond to diverse shocks and disruptions but also identifies strategic entry points to strengthen resilience through targeted investments and interventions. Specifically, it emphasises strategies that can enhance youth’s capacity to anticipate and absorb short- to medium-term shocks, while simultaneously building their ability to adapt and transform in response to longer-term challenges. Anchored in principles of climate justice, the framework seeks to address underlying power imbalances, promote youth agency, and support more equitable and inclusive adaptation pathways.

The proposed framework (see figure 8) adopts a systems-thinking approach and utilises a climate justice lens to enhance youth resilience in the agri-food systems of the Global South. It recognises that youth, particularly those from marginalised communities, face disproportionate exposure to climate-related shocks due to historical and structural inequalities. As such, within the proposed strategies and interventions, the framework emphasises equitable, inclusive, and participatory strategies across four interconnected levels (individual, community, organisational, and system-wide). The proposed strategies and interventions aim to enhance youth’s capacity to anticipate and absorb short- to medium-term shocks such as economic disruptions, while simultaneously building their ability to adapt and transform in response to longer-term challenges such as climate change (see figure 8). The agri-food system operates across multiple levels; therefore, resilience efforts and strategies should address vulnerabilities and shocks at each level, with active support from stakeholders at those levels. The proposed framework aligns with climate justice principles by placing a central focus on youth agency, addressing power imbalances through equitable adaptation, and advocating for inclusive governance in agri-food systems.

At the individual level, resilience is enhanced through climate education, skills training, and income diversification. These efforts empower youth with knowledge of climate risks, practical agricultural skills, and multiple livelihood options to withstand shocks. For example, climate education could implement basic climate science education programs to inform youth about the principles of climate change, associated risks, and effective mitigation and adaptation strategies. Income diversification strategies could encourage youth to pursue multiple income streams, such as integrating crop cultivation with livestock production or agro-processing to improve financial stability during economic, climatic, or environmental shocks. These interventions aim to redress knowledge and resource gaps that limit youth adaptive capacity, particularly among those excluded from formal education or employment.

At the community level, the framework promotes collective resilience through intergenerational knowledge exchange, shared resource systems (e.g. seed banks), and the integration of indigenous and local knowledge. These strategies foster locally grounded, culturally relevant adaptation that empowers youth as agents of change within their communities. For example, intergenerational partnerships could be strengthened by supporting mentorship programs that connect experienced farmers with youth, facilitating knowledge transfer and fostering innovation. Climate-smart practices could be supported by raising awareness and promoting the adoption of low-cost, accessible, and environmentally friendly agricultural practices, emphasising indigenous knowledge and crops to enhance resilience to local climate challenges.

At the organisational level, climate justice is operationalised through inclusive governance, regular vulnerability assessments, and targeted support for historically excluded groups, including young women, youth with disabilities, and Indigenous youth. This ensures that resilience-building efforts are responsive to diverse needs and lived experiences, and that equitable access to support and opportunities is ensured across the agri-food system. For example, fostering collaboration among local governments, NGOs, humanitarian organisations, and civil society to implement climate-sensitive approaches that enhance youth adaptive capacity.

Together, these levels support youth in anticipating, withstanding, and adapting to shocks over time while creating space for them to influence and lead systemic change. At the system level, the framework calls for transformative policy and institutional change that addresses the root causes of youth vulnerability and exclusion from climate decision-making. It advocates for redistributive approaches that shift power and resources toward youth-led and community-driven solutions. By embedding climate justice principles across all levels, this framework moves beyond technical adaptation to promote structural transformation, ensuring that youth are not only resilient to climate shocks but also active participants in shaping equitable and sustainable food systems futures.

## 6. Implications for policy, practice and future work

**Figure 9.**
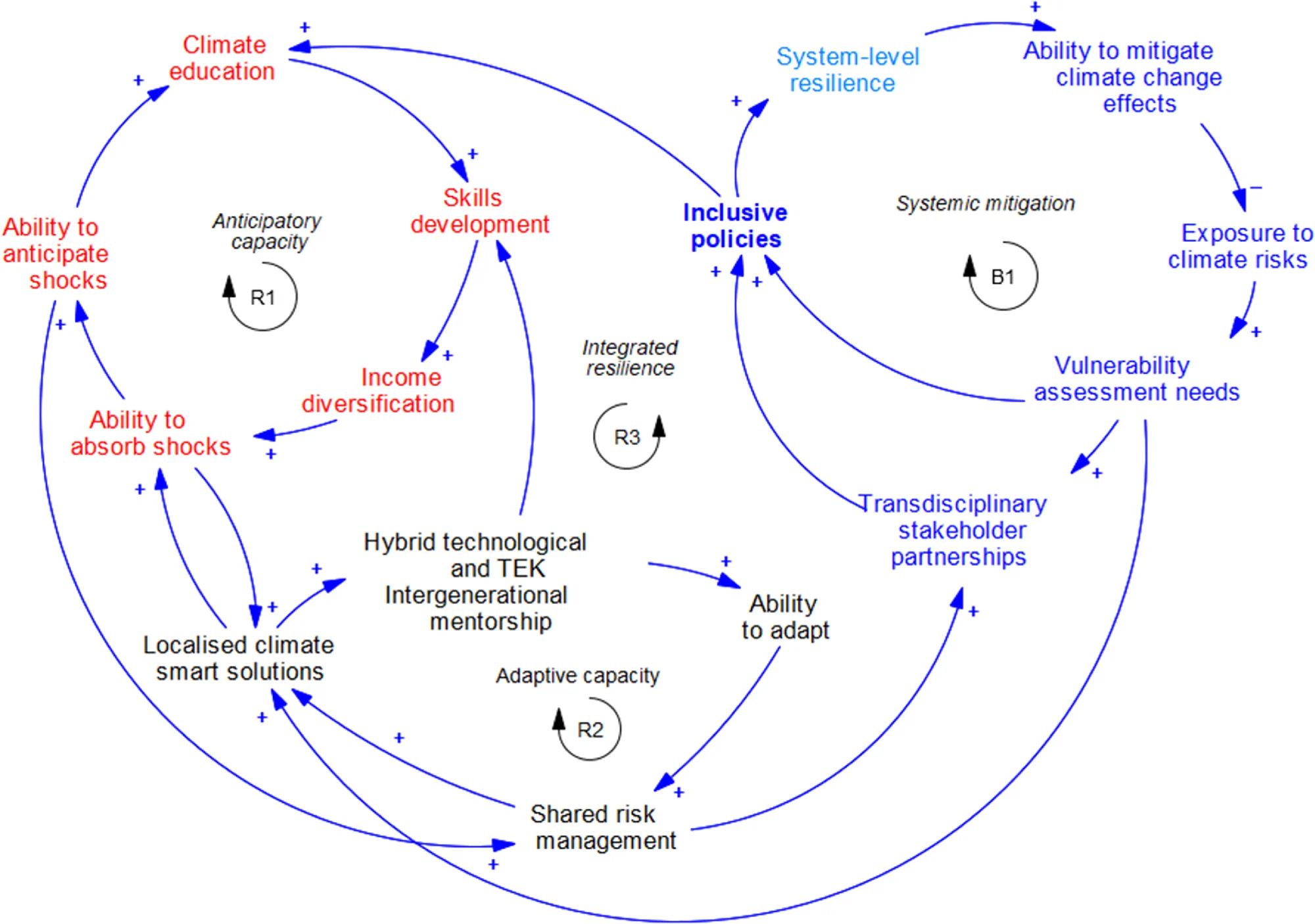
A causal loop diagram that maps the dynamic interactions between individual, community, organisational-level and systems-level interventions that influence youth resilience. The loops’ names are author-defined labels which describe feedback structures proposed in the conceptual framework. R1 (Anticipatory capacity): Climate education → Skills development → Income diversification → Ability to absorb shocks → Ability to anticipate shocks → Climate education. R2 (Adaptive capacity): Ability to adapt → Shared risk management → Localised climate-smart solutions → Hybrid technological and TEK Intergenerational mentorship → Ability to adapt. B1 (Systemic mitigation): Ability to mitigate climate change effects → Exposure to climate risks → Vulnerability assessment needs → Transdisciplinary stakeholder partnerships → Inclusive policies → System-level resilience → ability to mitigate climate-change effects. R3 (Integrated resilience): Climate education → Skills development → Income diversification → Ability to absorb shocks → Localised climate-smart solutions → Hybrid technological and TEK Intergenerational mentorship → Ability to adapt → Shared risk management → Transdisciplinary stakeholder partnerships → Inclusive policies → Climate education.

The CLD (see figure 9) visually maps the dynamic interactions between the factors outlined in the conceptual framework, showing how different individual, community, and organisational interventions interact to influence and reinforce youth resilience in agri-food systems. At the individual level, resilience-building focuses on climate education, skills development, and income diversification. In the CLD, these strategies enhance youth income stability, especially in the face of shocks, strengthening their adaptive capacity and overall resilience. At the community level, strategies such as shared risk management and localised climate-smart solutions are key drivers of adaptive capacity, particularly in the face of increasing climate variability, economic shocks, and systemic vulnerabilities. These approaches build collective resilience and reduce exposure to climate-related risks. Stakeholder partnerships foster an enabling environment at the organisational level through targeted institutional support, inclusive policies, and structural interventions. These elements support systemic change and strengthen the youth’s mitigation capacity over time by promoting long-term shifts towards resilient agri-food systems.

This CLD reflects core systems-thinking principles by illustrating the interconnectedness and feedback loops among individual-, community-, organisational-, and agri-food system-level actions (nested/micro systems) in shaping youth resilience. It highlights how interventions at one level reinforce outcomes at other levels, demonstrating a dynamic, non-linear system. In addition, it also emphasises leverage points such as adaptive capacity and enabling environments, where targeted actions can generate systemic change.

Moreover, the overarching theme within United Nations (UN) initiatives, such as the United Nations Framework Convention on Climate Change, promotes meaningful youth participation in climate-related policies and actions. This involves creating platforms for young people to contribute to climate discourse, ensuring their voices influence decision-making processes, and supporting youth-led climate initiatives. For example, guidelines and examples are outlined in the Youth Engagement Playbook for Cities (C40 Global Youth and Mayors Forum, 2021) and the checklist for decision-makers and practitioners on the youth-inclusive Nationally Determined Contributions process (Kaim 2023). Moreover, the recently adopted Pact for the Future: Action on Youth and the Declaration on Future Generations (United Nations 2024) call for enhanced youth participation in climate action at national and international levels to secure a better future for all and youth’s social and economic development to fulfil their potential and secure decent quality employment. By integrating multiple strategies for capacity building and youth involvement across multiple levels, this framework emphasises the importance of fostering robust resilience among youth in the Global South’s agri-food systems, reducing their vulnerability to shocks and stresses.

## 7. Strengths and limitations

The results of this study should be considered in light of certain limitations inherent in the methodology. Firstly, based on the study’s conceptual framing and definitions, the search strategy was limited to literature that uses the specific terminology. Second, some of the search terms used to identify relevant literature are broad and widely used in the broader literature on shocks and vulnerabilities in the food system. This is because, for broad, interdisciplinary topics like this one, narrower or more abstract search terms may miss relevant studies, especially when terminology varies across fields. This approach helps retrieve vast literature. However, this should consider the trade-offs between breadth and depth, as a lack of specificity can yield many irrelevant studies. Secondly, the articles included in the review were studies conducted in LMICs, which aligned with the objectives and interests of the study. Research conducted in high-income countries (HICs) was excluded from this analysis. For future research, we encourage broadening the scope of studies to include both LMICs and HICs, enabling comparisons across regions, additional analyses of inequalities and disparities within regions, and lessons that could be shared. Thirdly, although the current review identifies critical insights into youth resilience and engagement in agri-food systems, our evidence base is limited, even in LMIC contexts, and it is not diverse in the types of shocks youth may experience. Several critical dimensions that shape the realities of youth in LMIC are underrepresented, including gender constraints, informal agri-food systems where many young people derive their livelihoods, and displaced youth, whose vulnerability and coping strategies differ significantly from those of settled populations. These gaps underscore the need for more inclusive, context-sensitive research on youth in agri-food systems, emphasising the importance of climate justice and inclusive representation in shaping agri-food system responses to shocks and vulnerabilities.

## 8. Conclusion

The review sought to identify youth resilience to disruptions or shocks within the agri-food system. The literature highlighted gaps in knowledge addressing the main subject of the study. While much is said about the topic, most of it is rhetoric, as robust evidence does not support it. More empirical evidence is needed to identify how youth anticipate, cope with, and/or adapt to shocks within the agri-food system. Much of the literature examined focused on describing the impacts of shocks on the agri-food system and did not examine coping or adaptation strategies. In instances where this was done, such data is not disaggregated to show youth as a specific category. In addition to undertaking risk and vulnerability assessments focused on the agri-food system, there is a need to collect disaggregated data on gender and youth (including youth living with disabilities) for monitoring and evaluation.

Although not the focus of the current study, the systematic review process revealed that the evidence base is highly polarised towards youth barriers to participation in the agri-food system. They lack access to credit and finance, agency, land and water, as well as information and other support mechanisms. Within the context of this study, this shows that youth are a marginalised group in society, in general, and in LMICs, in particular. Additionally, this highlights that youth are highly vulnerable to shocks within the agri-food system as they have inherently low adaptive capacity. Despite this, youth have specific strengths and abilities to cope with shocks. This includes social networks, small investments, being open to alternative employment, and migration. Through these, young people can navigate and respond to diverse shocks and disruptions within the agri-food system. The CLD highlights the key drivers of long-term youth resilience in agri-food systems: adaptive capacity, shared risk management, and institutional support, demonstrating how coordinated interventions across individual, community, and organisational levels can generate systemic change. As such, these provide a basis for building incremental adaptation and resilience; however, this needs to be well planned and coordinated to avoid trade-offs that may arise from strategies such as migration.

The disparities in evidence regarding youth participation in agri-food systems and youth resilience in agriculture highlight important knowledge gaps and future directions. First, there is a need to consolidate information on youth participation in agriculture, focusing on synthesising the barriers and categorising them into social, economic, cultural, environmental, and other relevant categories. Second, these should be differentiated by region to address the matter of relevance to context and appropriate scale. Third, this would provide a basis for youth-focused risk and vulnerability assessments within agri-food systems. Lastly, the outcomes would inform regionally differentiated adaptation strategies that can be mainstreamed across policies and programmes to increase youth participation in the agri-food system. This will ensure that youth participation in agri-food systems brings about the desired transformation and resilience. Building youth resilience in the agri-food system is not only a development imperative but a matter of climate justice. Ensuring that youth, especially those most marginalised, are equipped to adapt and thrive in the face of climate shocks is essential for a just and sustainable future.

## Data Availability

The data that supports the findings of this study are openly available in the supplementary files of this article. Supplementary data 1 available at https://doi.org/10.1088/2976-601X/ae9f61/data1.
